# Lorenz–Gini method for quantitative assessment of particle distribution in composite microstructures from SEM feature areas

**DOI:** 10.1016/j.mex.2026.104141

**Published:** 2026-09-02

**Authors:** Festus Ben

**Affiliations:** a Centre for Nanoengineering and Advanced Materials, Department of Metallurgy, University of Johannesburg, Johannesburg, South Africa; b Centre for Advanced Materials Research and Development, Department of Physics, Federal Polytechnic Ede, Ede, Nigeria

**Keywords:** ImageJ, Scanning electron microscopy, Image segmentation, Feature extraction, Area distribution, Cumulative distribution, Inequality metric, Microstructure quantification, Particle size analysis, Digital image analysis

## Abstract

Quantitative evaluation of reinforcement dispersion in composite microstructures is still often based on qualitative interpretation of scanning electron microscopy (SEM) images, particularly in agro-waste-reinforced MMC studies. This method presents a statistical approach for describing particle distribution using Lorenz curves and the Gini coefficient derived from segmented microstructural data. Particle areas obtained from image analysis are used to construct cumulative distributions. This enables quantitative assessment of inequality in the projected-area contribution of particles used to support reinforcement-dispersion evaluation. The approach provides a reproducible framework for comparing projected particle-area contribution patterns across samples without relying on subjective visual interpretation. The method is applicable to particulate-reinforced systems where feature size distributions can be extracted from micrographs.

The study:

• Converts segmented microstructural features into cumulative distributions for dispersion analysis.
• Uses Lorenz curves to represent distribution patterns of reinforcement phases.
• Applies the Gini coefficient as a scalar metric for comparing dispersion across samples.

## Graphical abstract

**Figure:**
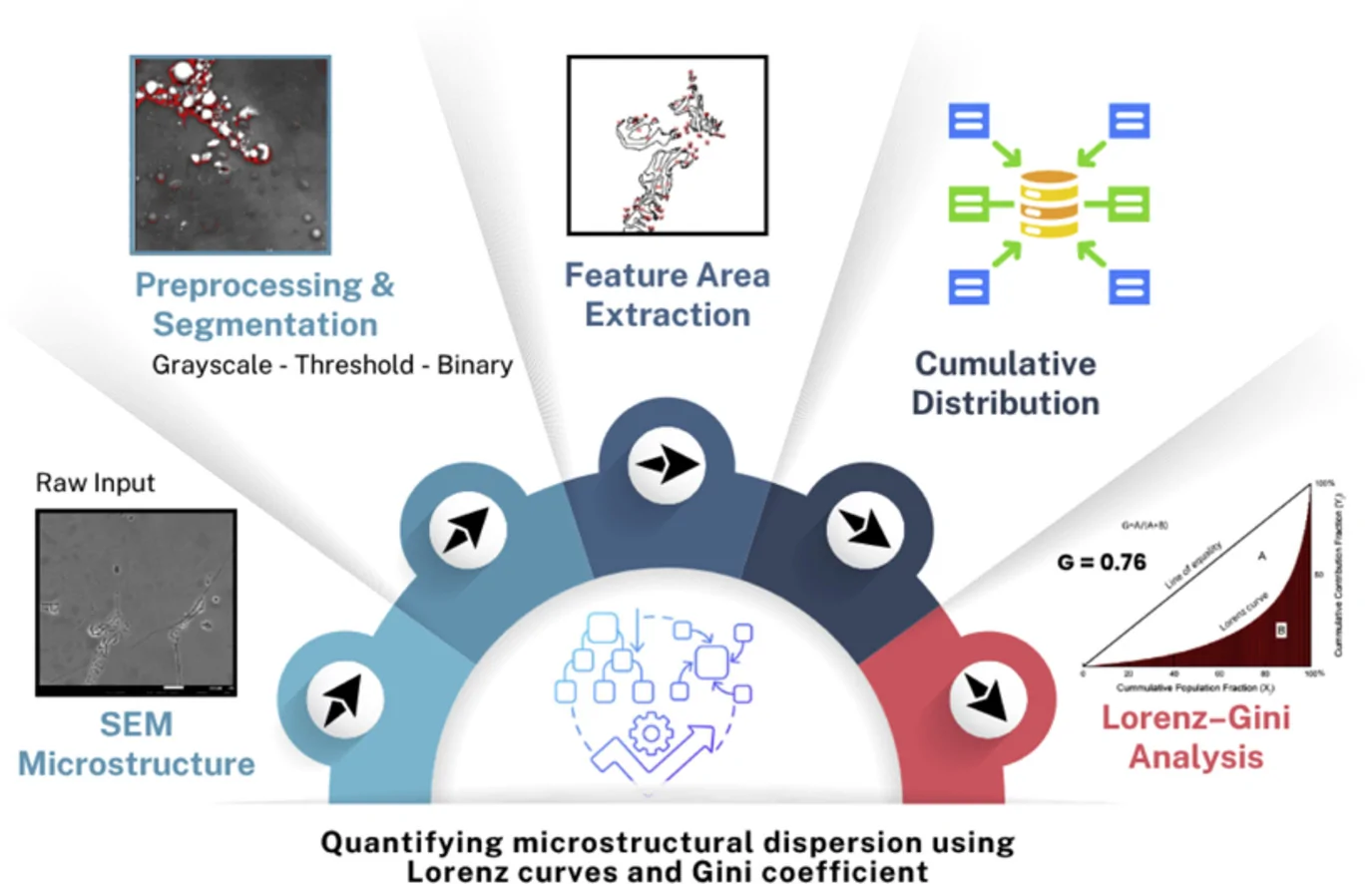

**Specifications table**

**Table:** 

**Subject area**	Materials Science
**More specific subject area**	Microstructural characterization and image-based analysis of composite materials
**Name of your method**	Quantitative assessment of particle dispersion using Lorenz curves and Gini coefficient
**Name and reference of original method**	Lorenz, M. O., Methods of measuring the concentration of wealth, Publications of the American Statistical Association. 9 (1905) 209–219. Gini, C., Variabilità e Mutabilità, Contributo allo studio delle distribuzioni e delle relazioni statistiche. C. Cuppini, Bologna (1912). Ceriani, L., Verme, P., The origins of the Gini index, Journal of Economic Inequality. 10 (2012) 421-443. 10.1007/s10888-011-9188-x
**Resource availability**	SEM micrographs of composite materials ImageJ: https://imagej.net/ij/ Spreadsheet software: Microsoft Excel or OriginLab

## Background

Scanning electron microscopy (SEM) is widely used for microstructural characterization in materials science. This is due to its ability to resolve phase morphology [1], particle distribution [2], defects [3], and interfacial features [4]. In multiphase materials, SEM images are often used to support claims related to dispersion, homogeneity, and agglomeration [5]. These characteristics are particularly important in metal matrix composites (MMCs), high-entropy alloys (HEAs), and ceramic or polymer composites, where the distribution of reinforcements influences the interpretation of processing routes and material performance [[6], [7], [8]].

In agro-waste-reinforced MMCs, reinforcement distribution is still commonly described from micrographs using qualitative expressions such as homogenous, uniform, clustered, or agglomerated [9,10]. Recent studies on rice husk ash, coconut-derived ash, and other waste-derived reinforcements continue to use microstructural observations to support such descriptions [11,12]. Quantitative approaches for particle characterization are, however, well established. These include particle size distribution, nearest-neighbor measurements, interparticle spacing, and tessellation-based methods for spatial organization [2,13,14]. These approaches measure different aspects of a particle population. Selected methods and their distinction from the present approach are summarized in Table 1. However, they do not describe how particle contributions are distributed within the microstructure. In particular, they do not account for imbalance in feature contribution or represent cumulative contribution patterns, i.e., the presence of dominant features within a population

**Table 1:** Comparison of selected approaches used to characterize particle distribution in composite microstructures.

Approach	Primary input	Main quantity described	Spatial arrangement?	Main application	Ref.
Qualitative microstructural assessment	SEM/optical micrograph	Apparent uniformity, agglomeration, morphology, and particle distribution	Visual only	Commonly used to describe reinforcement dispersion in agro-waste MMCs	[13,15]
Ripley’s K Statistical/functional spatial methods	Particle coordinates	Scale-dependent clustering or spatial randomness	Yes	Characterizes spatial distribution over selected length scales	[14]
Distance-based methods	Particle centroid coordinates	Interparticle spacing, nearest-neighbor distance, free-path distance	Yes	Quantifies local spatial separation	[14]
Particle size distribution	Individual particle dimensions or projected areas	Frequency and spread of particle sizes	No	Quantifies particle-size population but not particle positions	[2]
Voronoi/Tessellation-based methods	Particle centroid coordinates	Local neighborhood and spatial organization	Yes	Quantifies spatial arrangement through Delaunay/Dirichlet or Voronoi partitions	[14]
Lorenz–Gini analysis	Individual projected feature areas	Inequality in cumulative projected-area contribution across the detected particle population	No	Provides graphical and scalar descriptors of particle-area contribution patterns from segmented micrographs	This study

Lorenz curves and the Gini coefficient are established statistical tools for describing cumulative distribution and inequality [[16], [17], [18]]. The methodological contribution of the present study is a reproducible workflow for applying these concepts to segmented SEM feature areas. Extracted particle areas are ordered and converted into cumulative particle-count and projected-area contribution fractions. These are used to construct Lorenz curves and calculate corresponding Gini coefficients. The resulting descriptor quantifies inequality in projected feature-area contribution across the detected particle population. It does not independently describe particle positions or interparticle spacing. The method, therefore, complements particle-size and coordinate-based spatial metrics rather than replacing them. It extends the earlier application of Lorenz-Gini analysis to AA6063-based agro-waste composites [19] by providing the detailed procedure required for reproducible implementation.

## Method details

### SEM image selection and acquisition

SEM micrographs are treated as statistical samples of the microstructure. Image consistency is important.

• Images should be acquired under stable conditions: Use the same detector mode for all samples (secondary electron or backscattered electron). Keep the accelerating voltage and working distance constant. Changes in these settings affect contrast and feature boundaries.
• Use a fixed magnification for all images: Recommended range is ×500 to ×1000. Lower magnification may miss fine features. Higher magnification may reduce the number of detectable features. Consistent magnification ensures comparable feature populations.
• Each image should contain a sufficiently large and representative feature population. Small feature populations should be avoided because Gini estimates can be affected by finite-sample bias, while the magnitude of this effect depends on the underlying feature distribution [20,21].
• To reduce sampling bias, analyze multiple fields of view. Use 3–5 SEM images per sample from different regions to mitigate location-specific bias.

### Image preprocessing and segmentation

**Fig. 1:**
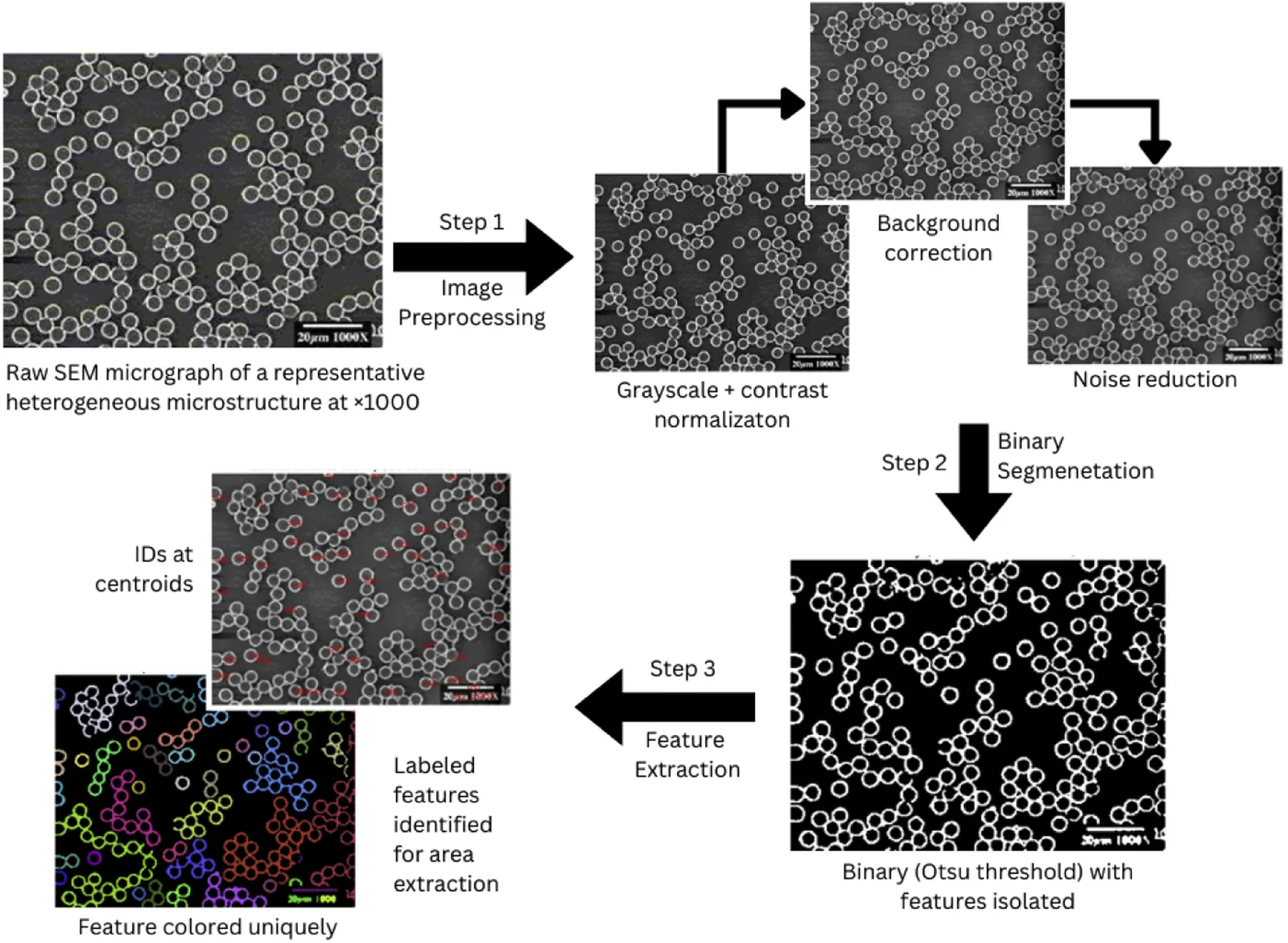
Representative workflow for SEM image preprocessing and feature segmentation. The procedure includes raw image acquisition, grayscale preprocessing for contrast normalization and noise reduction, binary segmentation of microstructural features, and feature labeling for subsequent extraction of projected area.

The overall workflow is illustrated in Fig. 1. It shows the sequence of image preprocessing, segmentation, and feature extraction steps used prior to Loren-Gini analysis.

**Fig. 2:**
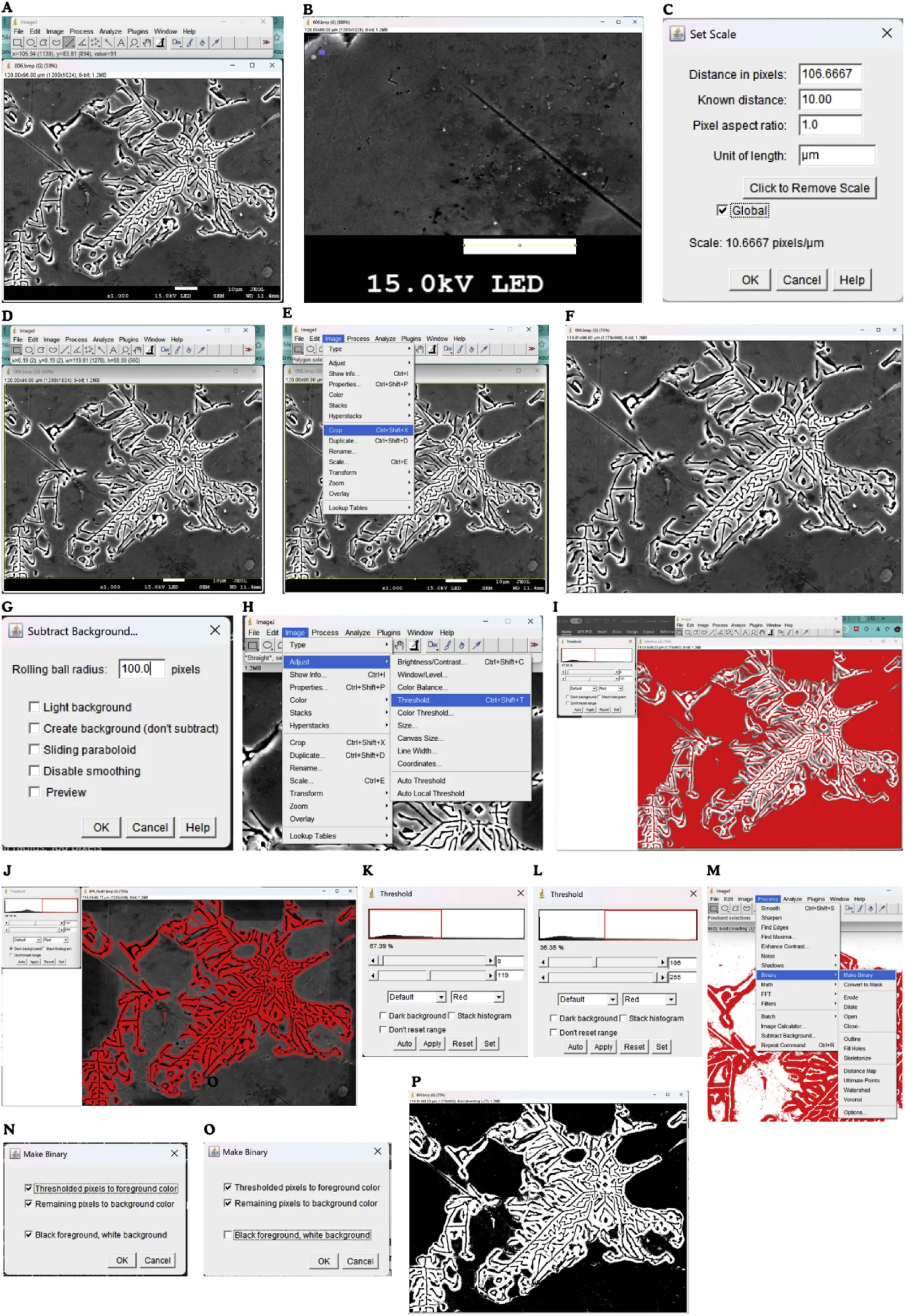
Workflow for SEM image preprocessing and segmentation using ImageJ. (A) Import of SEM micrograph. (B) Selection of scale bar using line tool. (C) Scale calibration using “Set Scale” dialog. (D) Selection of image region for cropping. (E) Crop operation. (F) Cropped image without the annotation band. (G) Background subtraction using rolling ball method. (H) Threshold interface. (I) Selection of the thresholding method. (J–L) Adjustment of threshold levels using histogram sliders. (M) Optimized threshold selection. (N) Access to binary conversion. (O-P) Binary conversion settings. (Q) Final binary image for particle identification.

The detailed implementation of the preprocessing and segmentation workflow using ImageJ is presented in Fig. 2.

• Import SEM images into ImageJ (Fig. 2A).
• Calibrate the image scale using the scale bar embedded in the original SEM image. Zoom in on the scale bar using the magnifying-glass tool, then draw a straight line across its known length with the line tool (Fig. 2B).
• Select *Analyze → Set Scale* to open the calibration dialog (Fig. 2C). The *Distance in pixels* is populated automatically from the drawn line. Enter the corresponding *Known distance* indicated on the SEM image (e.g., 10 µm), set the *Unit of length* to µm, and retain a pixel aspect ratio of 1.0. Select *Global* only when the same calibration is applicable to all images processed within the dataset. Click *OK* to apply the calibration.
• Return the image to a suitable viewing magnification and remove the SEM information footer. Using the rectangle-selection tool, select only the image field while excluding the lower band containing the scale bar, accelerating voltage, detector information, magnification, and other acquisition details (Fig. 2D).
• Select *Image → Crop* (Fig. 2E) to obtain the analysis field shown in Fig. 2F. The same cropped image dimensions should be maintained across comparable fields wherever the acquisition format permits.
• Convert to grayscale if required. Click *Image* → *Type* → *8-bit*. Subsequent intensity-based processing should be performed on the 8-bit image.
• Correct low-frequency background-intensity variation using *Process → Subtract Background* (Fig. 2G). During the development of the present workflow, rolling-ball radii ranging from 100 to 200 pixels were examined. Select a radius sufficiently large to suppress gradual background variation without altering the morphology or boundaries of the features of interest. A radius of 200 pixels satisfied this criterion for the SEM images analyzed in this study and was therefore maintained across the comparable image fields.
• Avoid unnecessary contrast or intensity manipulation after background correction. Preferably, leave all settings unchecked and adjust only the rolling ball radius. However, where adjustment is required, verify that it does not alter feature geometry, create artificial boundaries, or remove physically resolved features. The objective of preprocessing is to improve phase discrimination while preserving the morphology present in the original SEM image.
• Initiate segmentation using *Image → Adjust → Threshold* (Fig. 2H). Examine the grayscale histogram together with the red threshold overlay on the corresponding SEM image (Fig. 2I).
• Select Dark background in the Threshold window to establish the initial threshold orientation automatically. Leave “Stack histogram” and “Don’t reset range” unchecked for individual SEM images (Fig. 2J). This places the selected threshold towards the brighter feature population and reduces reliance on manual slider positioning.
• Inspect the histogram and corresponding preview, then verify that the selected range excludes most matrix/background regions while retaining the intended particle features. Where the automatic threshold produces artificial bridges or obvious matrix inclusion, adjust the lower boundary minimally until the segmentation better matches the visible morphology of the original SEM image. The intermediate threshold states illustrated in Fig. 2K → Fig. 2L demonstrate this refinement process.
• Stop the adjustment when further movement of the threshold boundary begins to remove genuine feature regions or alter their visible morphology. The resulting histogram position, rather than a fixed threshold number, defines the segmentation criterion. Consequently, the numerical threshold value may differ among independent SEM fields because their grayscale intensity distributions and histogram profiles can differ, even when the same preprocessing and segmentation procedure is applied.
• Compare the final threshold preview directly with the original SEM morphology before accepting the segmentation. Features that are visibly separated in the original image should remain separated wherever the grayscale information permits their distinction. If the threshold creates artificial bridges between neighboring features, adjust the boundary minimally until the visible separation is restored without unnecessarily removing genuine feature regions.
• Click *Apply* only after the histogram-based threshold selection and morphology check are complete.
• Convert the thresholded image to binary. Click *Process* → *Binary*→ *Make Binary* (Fig. 2M). Configure the binary conversion so that the threshold-selected pixels constitute the foreground and the remaining pixels constitute the background, as illustrated in Fig. 2N and 2O. The resulting binary image used for subsequent feature extraction is shown in Fig. 2P
• Apply additional noise filtering only where isolated pixel-scale objects remain after segmentation. Where required, use *Process → Noise → Despeckle* and inspect the result to confirm that isolated segmentation noise is removed without eliminating genuine small features. The filtering decision and settings should be applied consistently to image fields acquired and processed under comparable conditions.
• Use the resulting binary mask for connected-feature identification and subsequent particle analysis. Each spatially connected foreground object is treated as an individual segmented feature for extraction of its projected area in the subsequent *Analyze Particles* procedure.

### Feature extraction and data preparation

**Fig. 3:**
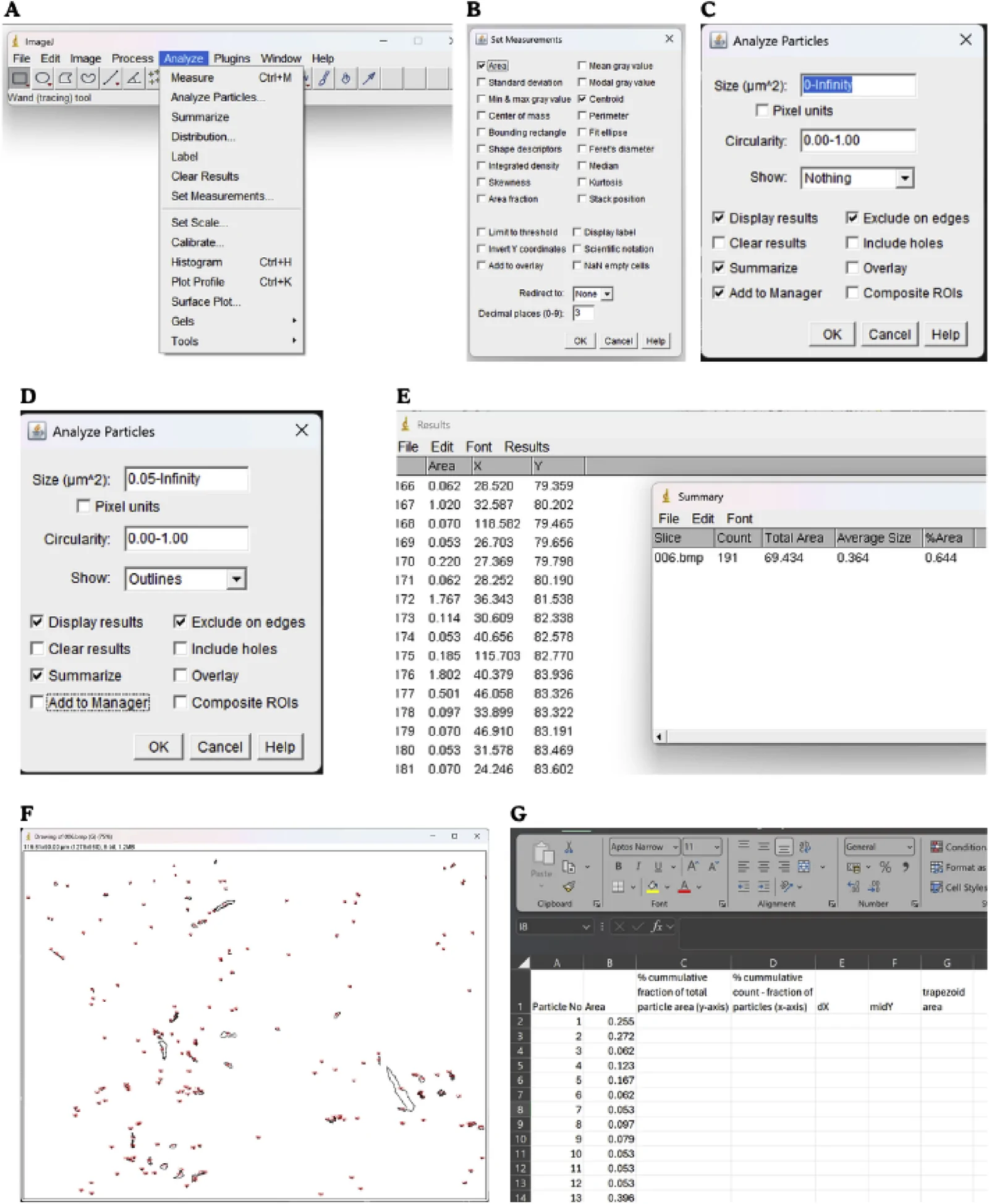
Feature extraction and data preparation using ImageJ and spreadsheet analysis. (A-B) Selection of measurement parameters, with area as the primary metric. (C-D) Particle analysis settings for feature detection and noise exclusion. (E) Extracted results table containing feature areas. (F) Visualization of detected features treated as individual population elements. (G) Transfer of data into spreadsheet software for sorting and subsequent Lorenz-Gini analysis.
• Use *Analyze → Analyze Particles* to extract the segmented feature data (Fig. 3C).
• Set the minimum particle-size cutoff to exclude residual objects below the physically resolved feature size and likely attributable to segmentation noise. The appropriate cutoff depends on image resolution and acquisition conditions. For the images analyzed in this study, *Size (µm^2)* was set to 0.05–Infinity. The same cutoff was maintained for images acquired under identical conditions. For other datasets, the cutoff should be selected with reference to image resolution and the smallest feature intended for quantitative measurement.
• Select ‘Outlines’ from the *Show* drop-down menu. Tick *Display Results, Summarize*, and *Exclude on Edges* (Fig. 3D). Apply the same settings to all comparable images.
• Inspect the generated particle-outline image against the segmented binary image to verify that the detected objects correspond to the features retained after segmentation. This provides a final visual quality-control step before exporting the quantitative data.
• Export the Results table (Fig. 3E) using *Edit → Select All → Copy*. The table contains the projected area measured for each detected feature.
• Treat each detected feature as one element of the statistical population (Fig. 3F), with its projected area representing its contribution to the total segmented feature area.
• Paste the copied Results table into a spreadsheet application such as Excel (Fig. 3G) for subsequent numerical processing.

• Set Measurements using *Analyze → Set Measurements* (Fig. 3A). Select ‘Area’ and, optionally, ‘Centroid’ if spatial information is required (Fig. 3B). Other measurement parameters are not required for the Lorenz–Gini computation.

### Macro-assisted implementation of the image-processing workflow

• The ImageJ macro provided in Supplementary File S2 implements the preprocessing, segmentation, and feature-extraction sequence described above. The macro is intended to standardize repeated processing while retaining user verification at stages where image-specific judgment is required.
• Open the raw SEM image in ImageJ using *File → Open*.
• Set the image scale from the SEM scale bar before running the macro, following the procedure described in Fig. 2B–C. Scale calibration is performed before cropping because the scale bar is located within the instrument-generated footer that is subsequently removed.
• Run the macro using *Plugins → Macros → Run...* and select *ImageJ_Macro_Lorenz_Gini_v2.ijm* from Supplementary File S2.
• The macro first checks the dimensions of the raw SEM image. For the validated image format used in this study (1280 × 1024 pixels), the upper 1280 × 960-pixel SEM region is retained automatically, and the lower 64-pixel instrument annotation band is removed. This eliminates operator-dependent variation in manual cropping.
• If the raw image does not match the validated 1280 × 1024-pixel format, the macro does not apply a fixed crop. Instead, it pauses and instructs the operator to manually define the SEM micrograph region, excluding the instrument annotation/footer band. This accommodates SEM images exported from different instruments or in different software formats.
• Enter the required preprocessing parameters when prompted. For the present validation, a rolling-ball radius of 200 pixels and a minimum feature area of 0.05 µm² were used. The optional *Despeckle* filter remains disabled unless noise filtering is required and is applied consistently to all comparable images.
• The macro converts the cropped image to 8-bit grayscale and applies rolling-ball background subtraction before opening the ImageJ Threshold window.
• In the Threshold window, select “Dark background” to establish the initial threshold orientation towards the brighter feature population. Leave “Stack histogram” and “Don’t reset range” unchecked for individual SEM images (Fig. 2J).
• Inspect the threshold preview against the original SEM morphology. Confirm that the matrix/background is largely excluded and that neighboring features that are visibly separated in the original image have not become artificially connected during segmentation. Where false bridges or obvious matrix inclusion occur, adjust the lower threshold boundary minimally until the segmentation better represents the visible feature morphology (Fig. 2K to 2M).
• Click *Apply* after the threshold and morphology checks are completed. The macro then proceeds to the feature-analysis stage using the defined measurement and particle-analysis settings.
• The macro records projected feature area as the principal measurement parameter, with centroid coordinates retained where required. *Analyze Particles* is executed using the specified minimum feature-area cutoff, with *Show → Outlines, Display Results, Summarize*, and *Exclude on Edges* selected (Fig. 3).
• Save the resulting binary mask and particle-outline image as part of the processing record. Export the ImageJ Results table containing the individual feature areas for subsequent cumulative-distribution, Lorenz-curve, and Gini-coefficient calculations.
• The macro standardizes the processing sequence but does not impose a fixed numerical threshold across different SEM images. Image-specific threshold values are permitted because grayscale distributions and histogram shapes differ across fields; reproducibility is instead maintained through a consistent thresholding rule, a fixed preprocessing sequence, and a morphology-verification criterion.

### Construction of cumulative distributions

• Let the extracted feature areas be represented as a dataset of nelements.
• Sort the dataset in non-decreasing order such that x_1_ ≤ x_2_ ≤ … ≤ x_n_.
• Compute the cumulative population fraction $X_{i}$ and cumulative contribution fraction $Y_{i}$ using Eqs. (1) and (2) (Fig. 3F).
  (1) $$P_{k}=\frac{k}{n},\;k=1,2,3,\ldots ,\;n\;$$
  (2) $$L_{k}=\frac{\sum_{i=1}^{k}x_{i}}{\sum_{i=1}^{n}x_{i}}\;$$
• Include the boundary conditions (0,0) when plotting. The final point must be (1,1) or (100,100) if expressed in percentage.
• The ordered pairs $\left(X_{i},Y_{i}\right)$ define the field-level cumulative distribution.

### Lorenz curve construction

**Fig. 4:**
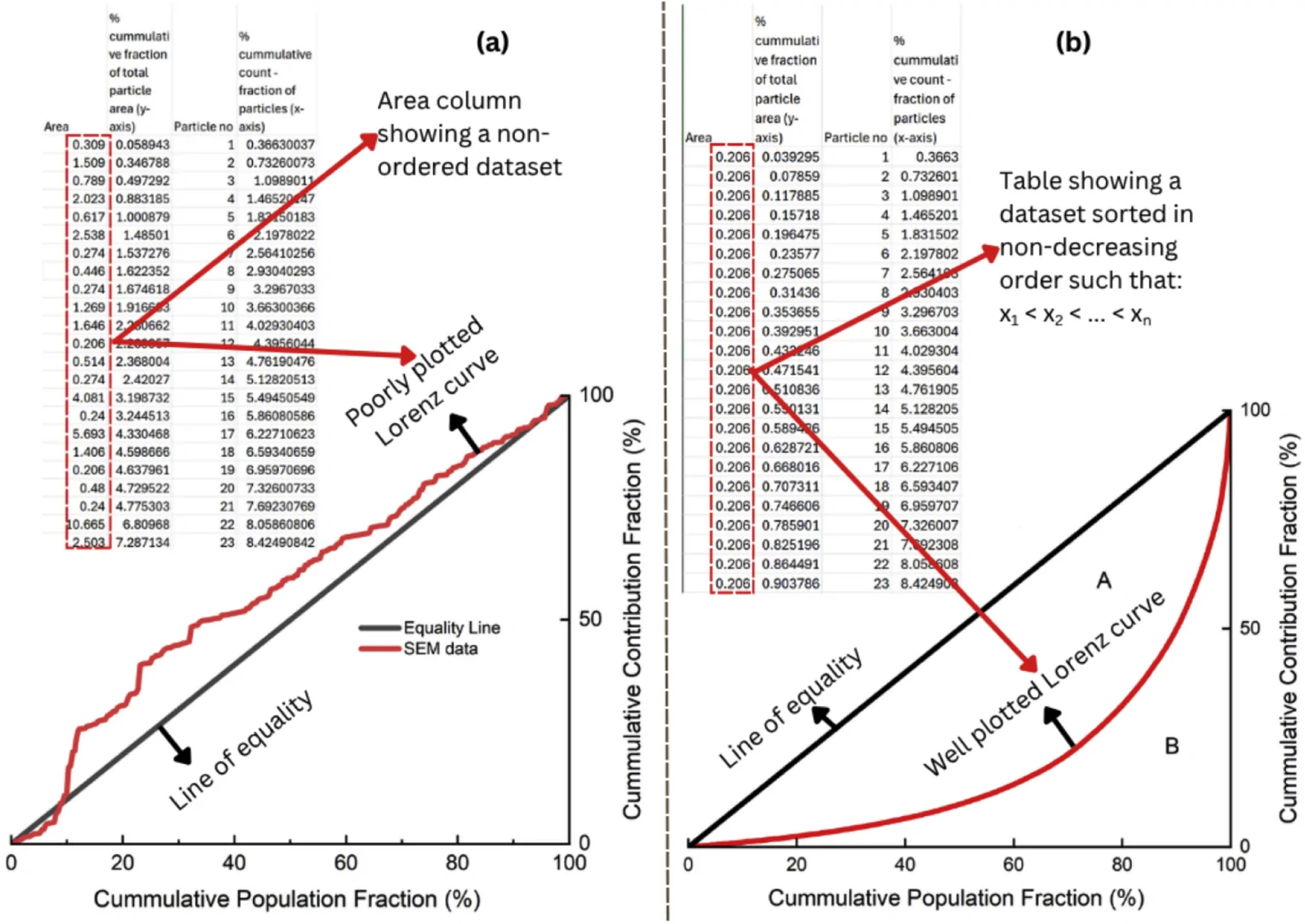
The influence of SEM particle area ordering on Lorenz curve generation; (a) unsorted data yielding an incorrect curve plot, and (b) the feature area sorted in ascending order, producing a valid Lorenz curve.
• For each independently analyzed SEM field containing segmented features, calculate the cumulative feature-population fraction, $X_{i}$, for the $i^{th}$ ordered feature using Eq. (3):
  (3) $$X_{i}=\frac{i}{n}\times 100\;$$
  where i=1,2,…,n is the rank of the feature after sorting and n is the total number of segmented features in the field.
• Calculate the corresponding cumulative feature-area contribution, $Y_{i}$, using Eq. (4):
  (4) $$Y_{i}=\left(\sum_{k=1}^{i}A_{k}/\sum_{k=1}^{n}A_{k}\right)\times 100\;$$
  where $A_{k}$ is the area of the $k^{th}$feature in the ordered dataset. This,$\;X_{i}$ represents the percentage cumulative feature population and $Y_{i}$ represents the percentage cumulative contribution of those features to the total segmented feature area. The spreadsheet formulas used to implement Eqs. (3) and (4) are provided in Supplementary File S1.
• Add the origin (0, 0) to the $X_{i}$ and $Y_{i}$ coordinates for each field. Each field-level cumulative distribution therefore extends from (0, 0) to (100, 100).
• Where multiple independent SEM fields are analyzed for the same material, differences in the number of detected features result in different increments along the cumulative feature-population axis. Consequently, the field-level cumulative-area values cannot be averaged directly on a row-by-row basis. To provide common population coordinates for comparison and averaging, define a common cumulative feature-population scale, X, from 0 to 100%. In the present implementation, 1% intervals were used (Supplementary File S1).
• For each field, determine the cumulative feature-area contribution at every common population fraction by linear interpolation between the two adjacent field-level coordinates that bound the required value of X. For $X_{1}\leq X\leq X_{2}$, where $\left(X_{1},\;Y_{1}\right)$ and $\left(X_{2},\;Y_{2}\right)$ are adjacent points on the field-level cumulative distribution, the interpolated cumulative feature-area contribution is given by Eq. (5):
  (5) $$Y\left(X\right)=Y_{1}+\frac{X-X_{1}}{X_{2}-X_{1}}\left(Y_{2}-Y_{1}\right)$$
  This procedure converts each independently analyzed field to the same 0–100% cumulative feature-population coordinates without altering its original cumulative feature-area distribution.
• Repeat the interpolation independently for all replicate SEM fields. At each common population fraction, calculate the mean cumulative feature-area contribution as in Eq. (6):
  (6) $$\bar{Y}\left(X\right)=\frac{1}{m}\sum_{j=1}^{m}Y_{j}\left(X\right)$$
  where mis the number of independently analyzed SEM fields and $Y_{j}\left(X\right)$ is the interpolated cumulative feature-area contribution of the field j at population fraction X. For the present validation, m=3.
• The complete spreadsheet implementation of the ordering, cumulative-fraction calculations, interpolation onto the common population scale, and averaging procedure is provided in Supplementary File S1 to facilitate reproducible implementation.
• Plot the common cumulative feature population (%), X, on the x-axis against the mean cumulative feature area (%), $\bar{Y}\left(X\right)$, on the y-axis to obtain the material-level mean Lorenz curve. Individual field-level Lorenz curves may similarly be obtained by plotting $Y_{i}$ against $X_{i}$ before interpolation.
• Include the line Y=X, extending from (0, 0) to (100, 100), as the line of perfect equality (Fig. 4b). Increasing deviation of the Lorenz curve below this line indicates progressively greater inequality in the contribution of segmented features to the total feature area.

• The feature number and corresponding feature-area measurements obtained from ImageJ are exported into a spreadsheet (Fig. 3G).
• Sort the individual feature-area values in non-decreasing order, from the smallest to the largest feature. Correct ordering is essential because the Lorenz curve represents the cumulative contribution of progressively larger features; unsorted feature-area data produce an incorrect cumulative distribution (Fig. 4a).

### Gini coefficient computation

**Fig. 5:**
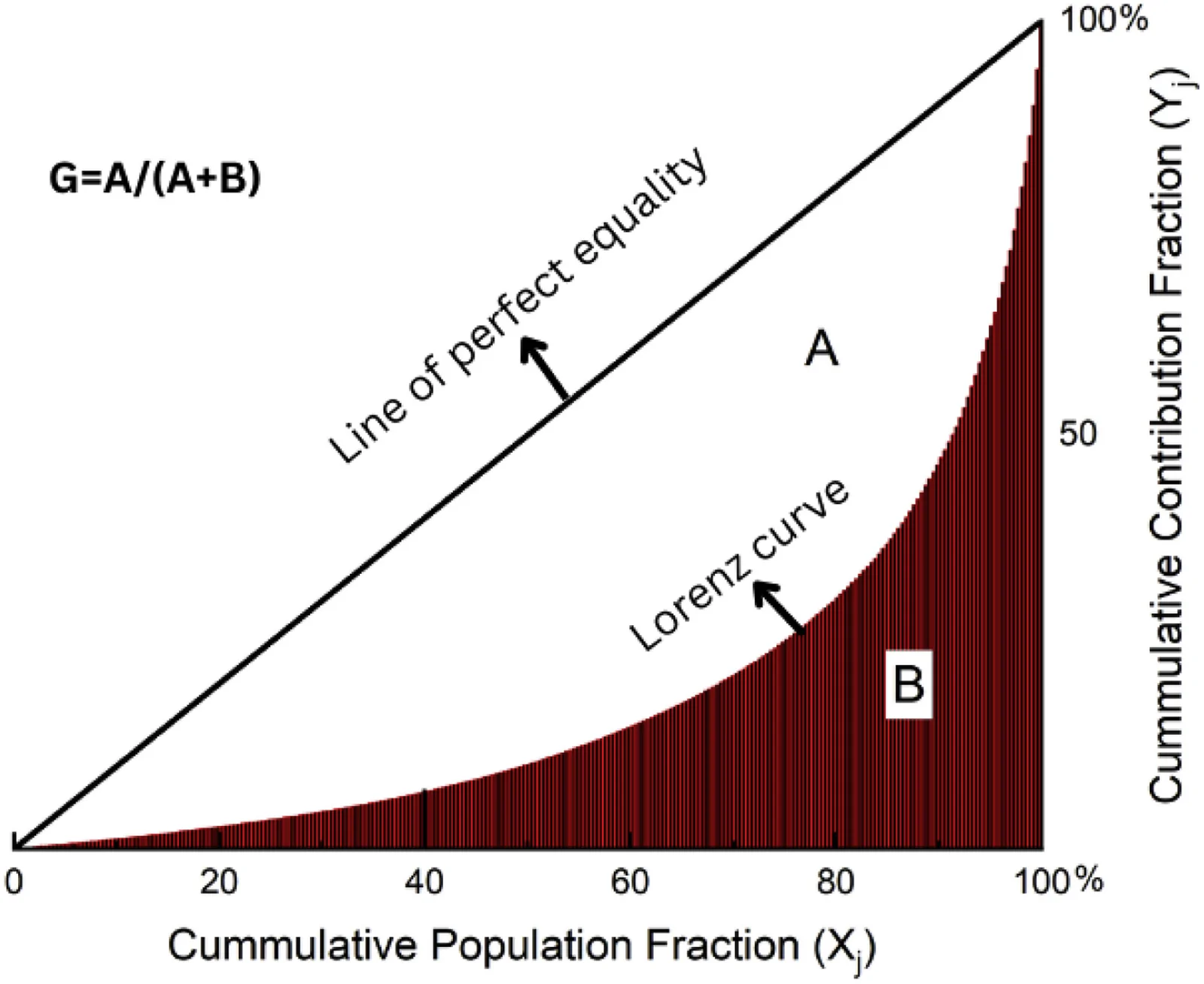
Trapezoidal approximation of the Lorenz curve for computing the Gini coefficient.
• Determine the area under the Lorenz curve, $A_{L}$, by trapezoidal integration. For a field containing n segmented features, including the boundary point $\left(X_{0},\;\;Y_{0}\right)=\left(0,\;0\right)$, calculation was done using Eq. (7):
  (7) $$A_{L}\;=\sum_{j=1}^{n}\left(X_{j}-X_{j-1}\right).\;\frac{\left(Y_{j}+Y_{j-1}\right)}{2}\;$$
  where $X_{j}$ and $Y_{j}$ are the cumulative feature-population and cumulative feature-area fractions, respectively. Fractions ranging from 0 to 1 are used in the computation.
• Calculate the field-level Gini coefficient using Eq. (8).
  (8) $$G=1-2A_{L}=1-\sum_{j=1}^{n}\left(X_{j}-X_{j-1}\right).\;\left(Y_{j}+Y_{j-1}\right)\;$$
  where G=0 represents equal feature-area contribution and increasing values towards 1 indicate progressively greater inequality in the contribution of segmented features to the total feature area.
• If required for datasets with limited feature counts, an optional finite-sample correction may be applied to the field-level Gini coefficient using Eq. (9):
  (9) $$G_{corr}=\frac{n}{n-1}\left(1-2A\right)\;$$
• Where n is the number of segmented features within the independently analyzed SEM field. This correction is optional and was not applied in the present study; all reported Gini coefficients were obtained directly from trapezoidal integration of the field-level Lorenz curves.
• For materials represented by replicate SEM fields, calculate the Gini coefficient independently for each field before deriving the material-level statistics. For m independent fields, calculate the mean Gini coefficient using Eq. (10) and the field-to-field standard deviation using Eq. (11):
  (10) $$\bar{G}=\frac{1}{m}\sum_{j=1}^{m}G_{j}\;$$
  (11) $$s_{G}=\sqrt{\frac{\sum_{j=1}^{m}{\left(G_{j}-\bar{G}\right)}^{2}}{m-1}}\;$$
  where $G_{j}$ is the Gini coefficient of the field j. The material-level result should be reported as $\bar{G}\pm s_{G}$. For the present validation, m=3 independent SEM fields.
• Where required, calculate the coefficient of variation to express the relative field-to-field variability using Eq. (12):
  (12) $$CV_{G}\left(\%\right)=\frac{s_{G}}{G}\times 100\;$$
• All calculations can be performed using spreadsheet software. The spreadsheet implementation used for trapezoidal integration and the field- and material-level Gini calculations is provided in Supplementary File S1.

• Calculate the Gini coefficient independently for each SEM field from the area under its field-level Lorenz curve (Fig. 5). The field-level cumulative feature-population and cumulative feature-area fractions described above are used for this calculation.

### Practical notes and reproducibility considerations

• Use the same preprocessing parameters for all images.
• Do not mix images taken at different magnifications.
• Ensure sufficient feature count per image.
• Check segmentation quality before analysis. Poor segmentation will affect results.
• Always sort features data before constructing cumulative distributions.
• Plot Lorenz curves together with Gini values for comparison.

### Method validation

To validate the proposed Lorenz-Gini framework, the method was applied to SEM microstructures of agro-waste-reinforced AA6063 composites previously reported in the literature [6]. The material system consists of monolithic AA6063 reinforced with individual cassava peel ash (CPA), coconut husk ash (CHA), and bean pod ash (BPA), all processed under identical conditions to eliminate compositional and processing variability.

Representative FESEM micrographs at ×1000 magnification were selected from the original dataset to ensure consistent imaging conditions. For each composite, three independent SEM fields of view acquired at ×1000 magnification from distinct microstructural regions were analyzed to reduce field-selection bias. These SEM images were processed using the same standardized image-processing and segmentation workflow. All images were processed using the methodology described in Section 2, including preprocessing, segmentation, and feature extraction in ImageJ. Supplementary File S2 provides the ImageJ macro used to standardize this processing sequence and facilitate reproducible implementation of the procedure in other studies.

**Fig. 6:**
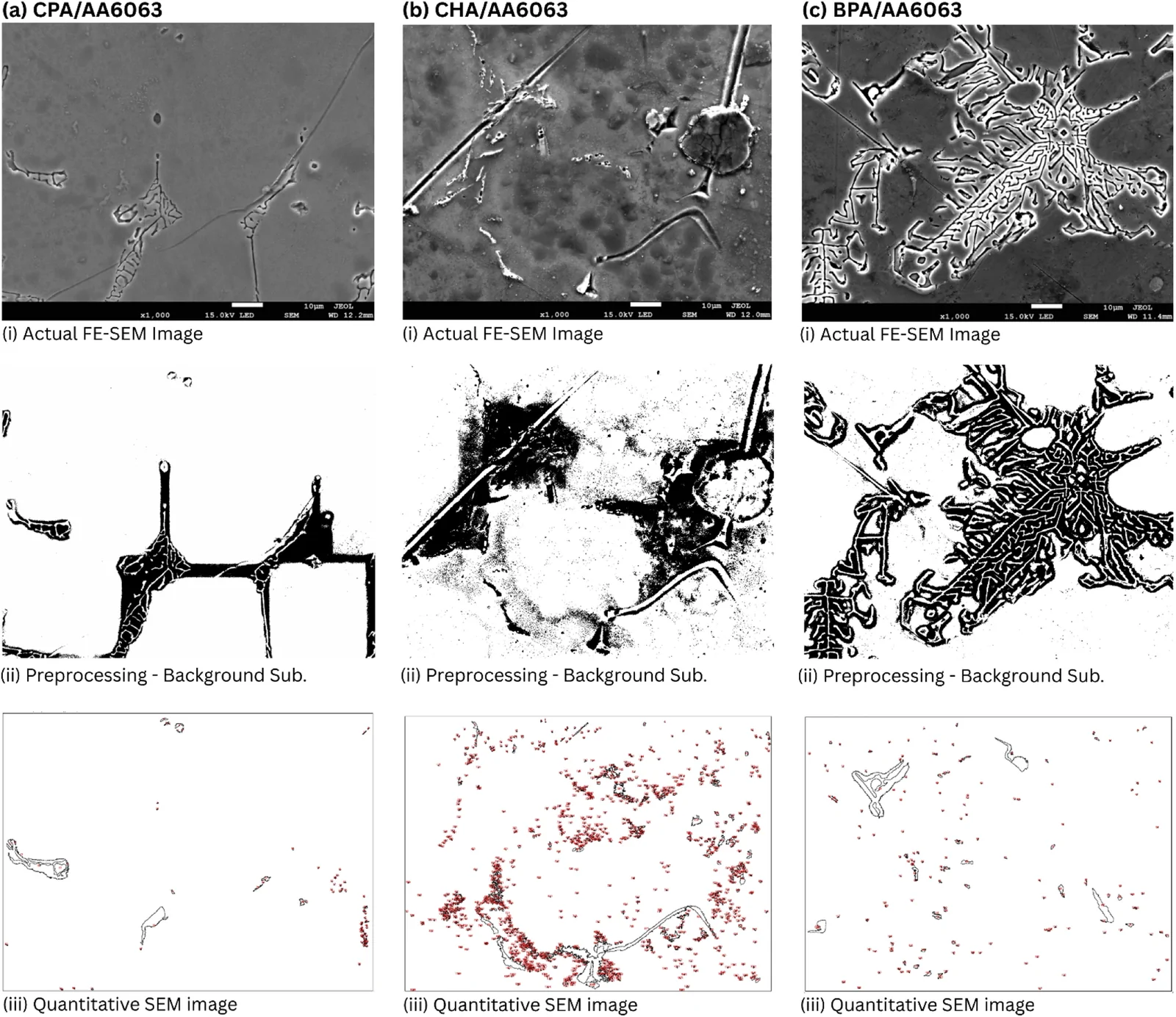
Representative ×1000 FESEM micrographs of monolithic (a) CPA-, (b) CHA-, and (c) BPA-reinforced AA6063 composites, showing (i) original image, (ii) background-corrected image after preprocessing, and (iii) segmented feature masks used for quantitative analysis.

The pre-processing and segmentation outputs are presented in Fig. 6, showing the original SEM micrographs, background-corrected images, and the corresponding segmented feature masks. Background subtraction was performed using a rolling radius of 200 pixels to enhance feature boundary detection prior to particle identification. Feature extraction yielded particle populations ranging from 65 to 105 for CPA, 70–1096 for CHA, and 51–146 for BPA, with corresponding total segmented areas of 103.11–175.17, 146.41–439.23, and 29.60–230.71 µm², respectively. The segmented area fraction also varied markedly among independent fields (CPA: 0.96–1.63%; CHA: 1.36–4.09%; BPA: 0.28–2.15%), reflecting field-dependent differences in the abundance and spatial distribution of extracted features rather than imposing a uniform segmentation outcome across micrographs. These variations demonstrate that the standardized workflow accommodated substantial field-to-field differences in feature abundance and segmented area while preserving a consistent image-processing procedure across all analyzed fields.

**Fig. 7:**
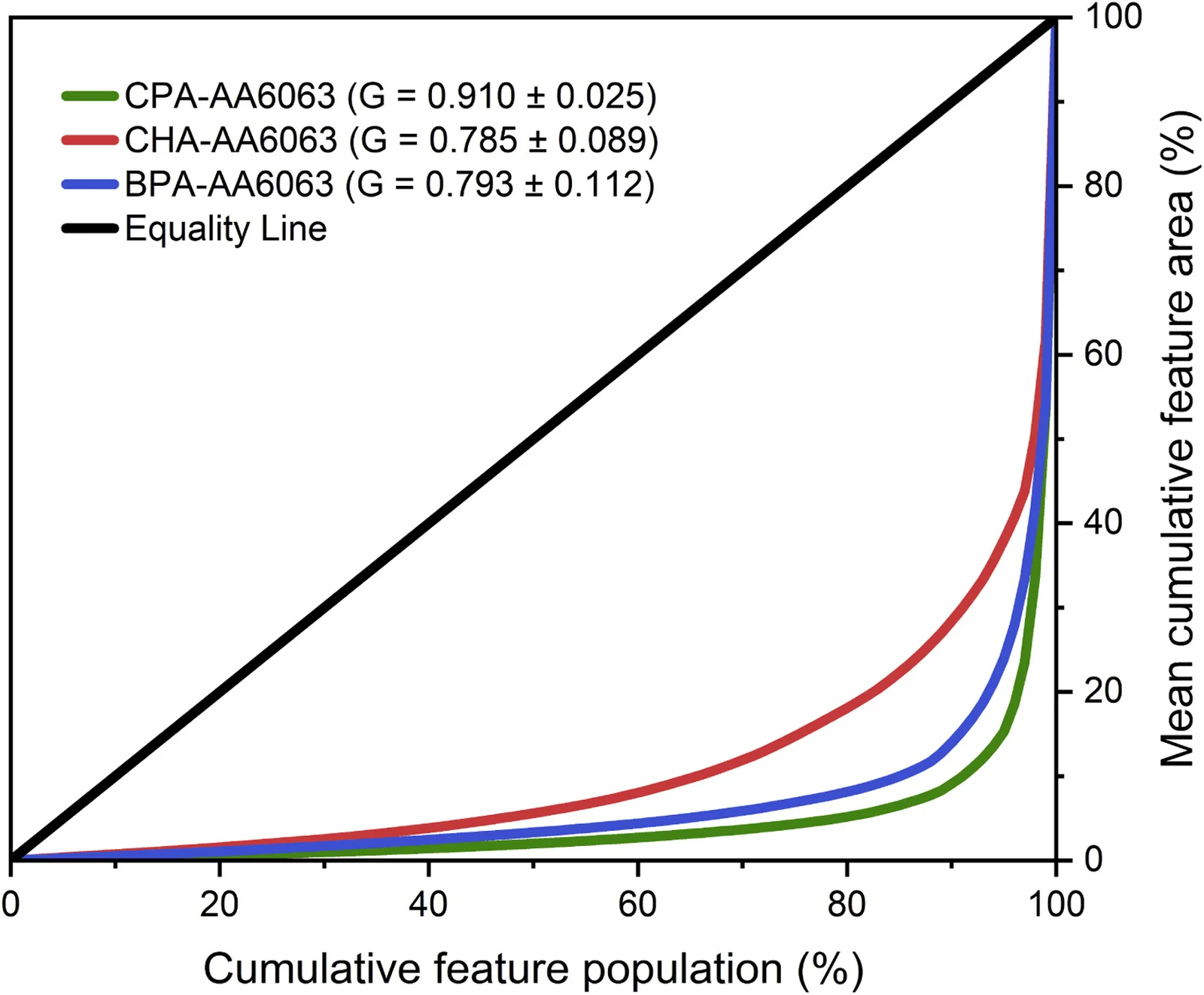
Mean Lorenz curves and corresponding Gini coefficients obtained from three independent SEM fields for AA6063 composites reinforced with CPA, CHA, and BPA particles. The curves represent the mean cumulative feature area at common cumulative feature-population fractions, obtained by interpolating the individual field-level Lorenz curves.

Cumulative population and feature-area contribution distributions were computed from the extracted feature areas, and the corresponding Lorenz curves are presented in Fig. 7. The feature-ordering procedure and Excel-based computations used to derive the Lorenz curves and Gini coefficients (Fig. 3G) are provided in Supplementary File S1. All samples exhibit deviation from the line of equality, indicating unequal contributions of the segmented reinforcement features to the cumulative feature area. Differences in the Lorenz-curve profiles among the composites demonstrate the method's sensitivity to variations in the distribution of feature-area contributions.

Since the three independently analyzed SEM fields contained different numbers of segmented features, their field-level Lorenz curves could not be averaged directly at the original particle-count coordinates. Each field-level curve was therefore linearly interpolated onto a common cumulative feature-population scale ranging from 0 to 100% at 1% intervals. At each common population fraction, the cumulative feature-area contributions from the three fields were averaged to construct the material-level mean Lorenz curve shown in Fig. 7. This procedure preserves the normalized cumulative distribution represented by each field while allowing fields with different feature counts to be compared and combined on a common basis. The resulting mean curves show distinct cumulative contribution profiles for CPA, CHA, and BPA, while the independently calculated field-level Gini coefficients provide the corresponding quantitative measure of feature-area inequality and its field-to-field variability.

The Gini coefficients obtained by trapezoidal integration of the Lorenz curves are also presented in Fig. 7. The Gini coefficient was calculated independently for each field, allowing field-to-field variability to be retained rather than obscured through pooling of the extracted features. Across the three independent SEM fields, mean Gini coefficients of 0.785 ± 0.089 for coconut husk ash, 0.910 ± 0.025 for cassava peel ash, and 0.793 ± 0.112 for bean pod ash were obtained. The corresponding coefficients of variation (CV) are 11.3%, 2.7%, and 14.1% for CHA, CPA, and BPA, respectively. Cassava peel ash, therefore, exhibits the highest mean feature-area inequality and the lowest field-to-field variability, whereas coconut husk ash and bean pod ash show greater variation among independently sampled fields. These results demonstrate that the workflow preserves genuine field-dependent microstructural variability while providing a consistent basis for quantifying and comparing cumulative feature-area distributions.

Previous descriptors of these microstructures were based on qualitative SEM interpretation and property-based inference [6]. The present analysis provides a quantitative representation of the distribution of feature contributions derived directly from segmented SEM data. These results demonstrate that the method:

• Produces stable cumulative distributions from SEM-derived feature data,
• Generates reproducible Lorenz curves under consistent preprocessing conditions, and
• Yields scalar metrics that distinguish differences in feature contribution across samples.

### Limitations

The proposed method is subject to the following limitations inherent to image-based microstructural analysis:

• The analysis is based on two-dimensional SEM projections of inherently three-dimensional microstructures. The projected feature area may not fully represent volumetric contribution or three-dimensional spatial connectivity. Analysis of multiple independent fields is therefore recommended to better represent within-specimen microstructural variability.
• The method quantifies inequality in projected feature-area contribution rather than all aspects of microstructural heterogeneity. Feature populations with similar area distributions but different spatial arrangements may therefore yield comparable Gini coefficients. The method does not independently quantify particle coordinates, interparticle spacing, or spatial clustering. Complementary descriptors, such as particle-size distribution, nearest-neighbor analysis, Voronoi-based descriptors, or spatial clustering metrics, may be required where these characteristics are of interest.
• The method depends on segmentation quality. Preprocessing operations such as background correction, threshold selection, noise filtering, and the minimum particle-area cutoff can influence feature identification and measured projected areas. Consistent processing criteria and verification of the segmented features against the original SEM image are therefore required for reproducible implementation.
• Touching or connected regions remaining after binary segmentation are treated as single segmented features in the present workflow. Consequently, the measured area of a connected feature may represent more than one physically distinct particle. Applying particle-separation procedures, such as watershed segmentation, would alter the resulting feature population and should therefore not be applied selectively when comparing images processed using the present procedure.
• Magnification, image resolution, and field-of-view selection influence the detected feature population and the resulting Lorenz–Gini estimate. Comparisons should therefore use consistent acquisition conditions and multiple independent SEM fields. Processing parameters validated for one imaging configuration, including cropping dimensions, rolling-ball radius, and minimum particle-area cutoff, should not be assumed to be universally transferable without verification.
• Multiple SEM fields obtained from the same specimen represent within-specimen subsampling rather than independent material replicates. Accordingly, field-to-field variability can be reported to assess the repeatability and representativeness of the image-based measurement, but material-level statistical inference requires independently prepared replicate specimens.

## Ethics statements

This study does not involve human subjects, animal experiments, or data collected from social media platforms*.*

## CRediT authorship contribution statement

**Festus Ben:** Conceptualization, Methodology, Data curation, Validation, Writing – original draft, Writing – review & editing, Software, Visualization.

## Declaration of interests

The authors declare that they have no known competing financial interests or personal relationships that could have appeared to influence the work reported in this paper.

## Data Availability

Data will be made available on request.

## Appendix Supplementary materials

Supplementary material *and/or* additional information [OPTIONAL] Supplementary File S1 – Excel file containing ordered feature area data, cumulative distributions, Lorenz curve construction, and Gini coefficient calculations for all analyzed composites. Supplementary File S2 – Reproducibility package containing the raw ×1000 SEM images, corresponding binary masks, detailed ImageJ processing parameters, and a semi-automated ImageJ macro for feature-area extraction.
